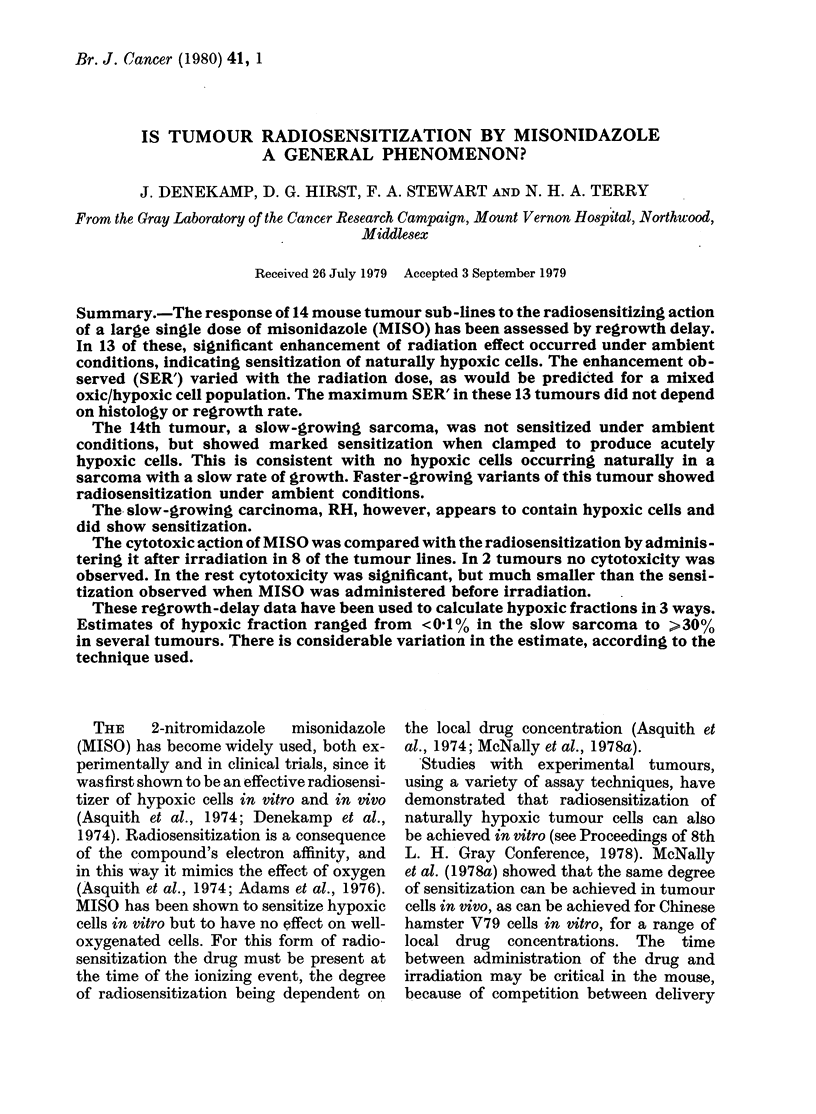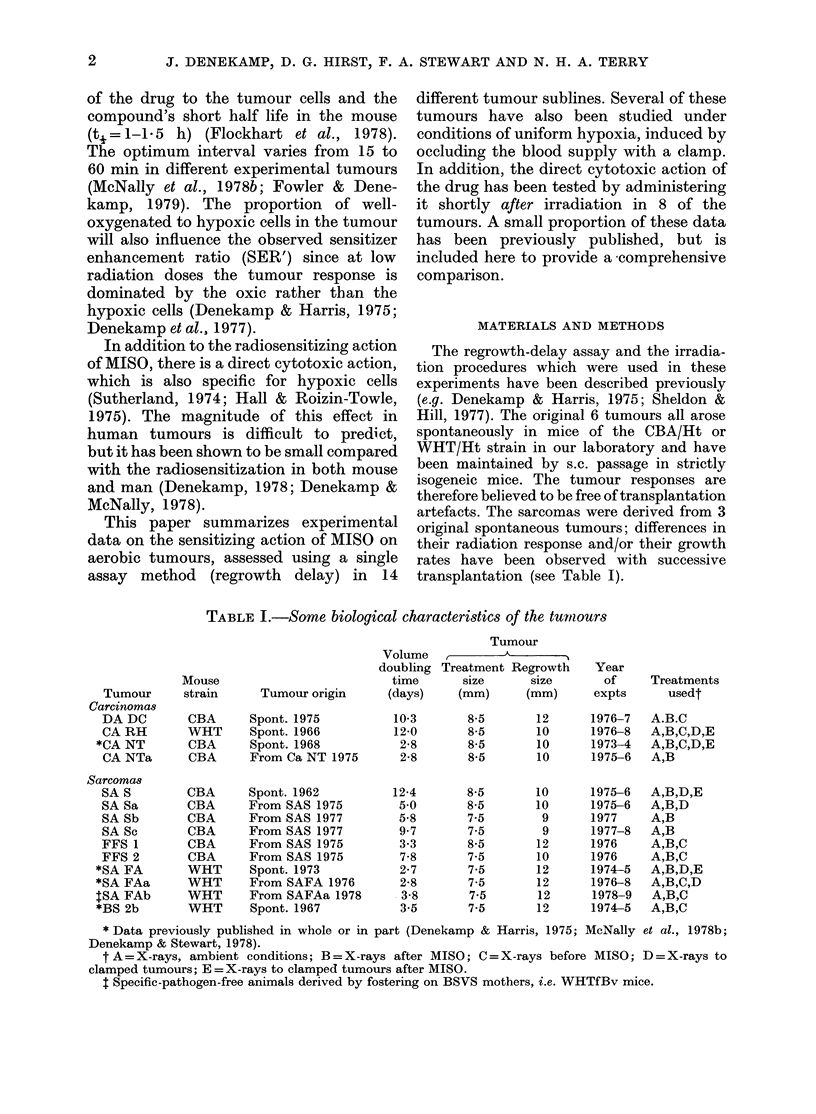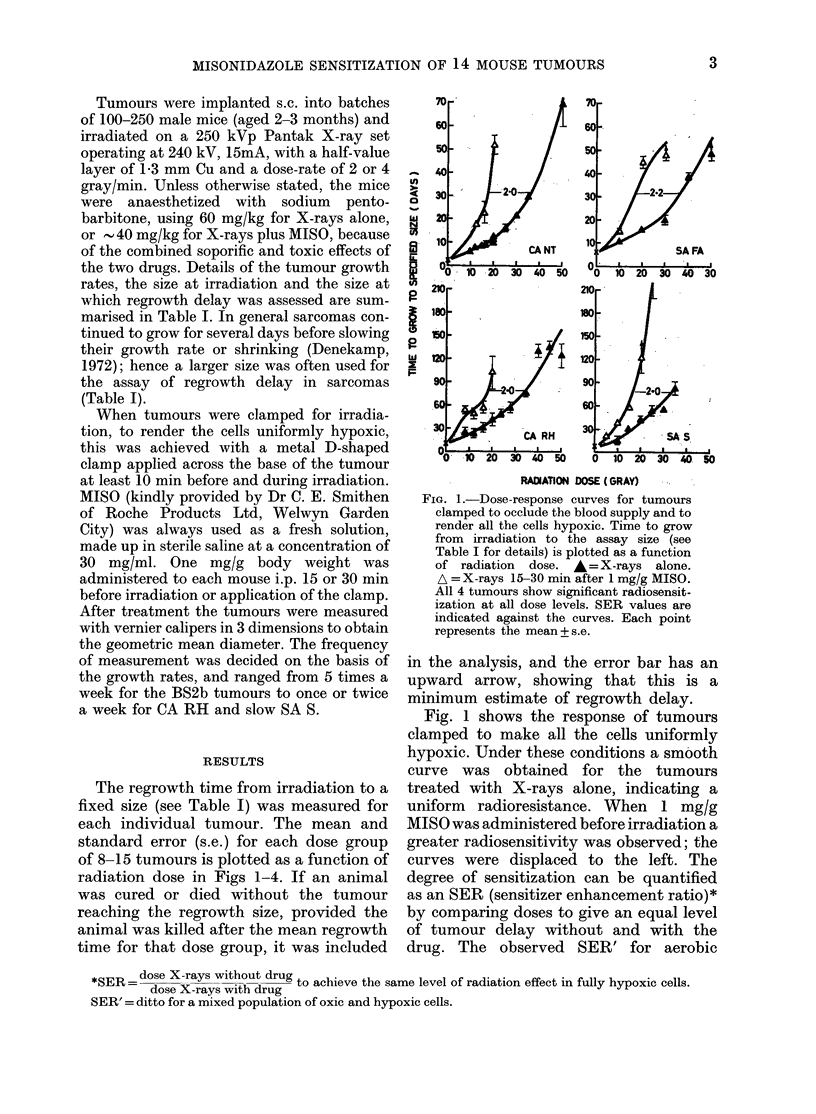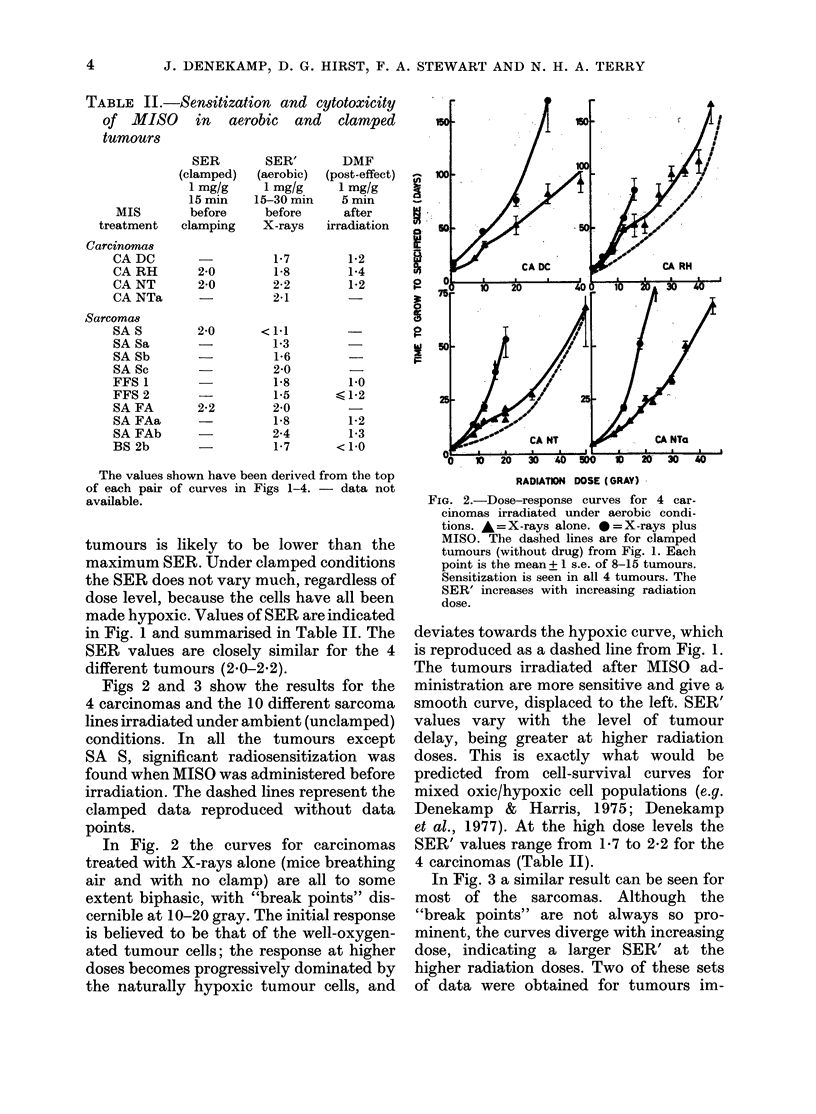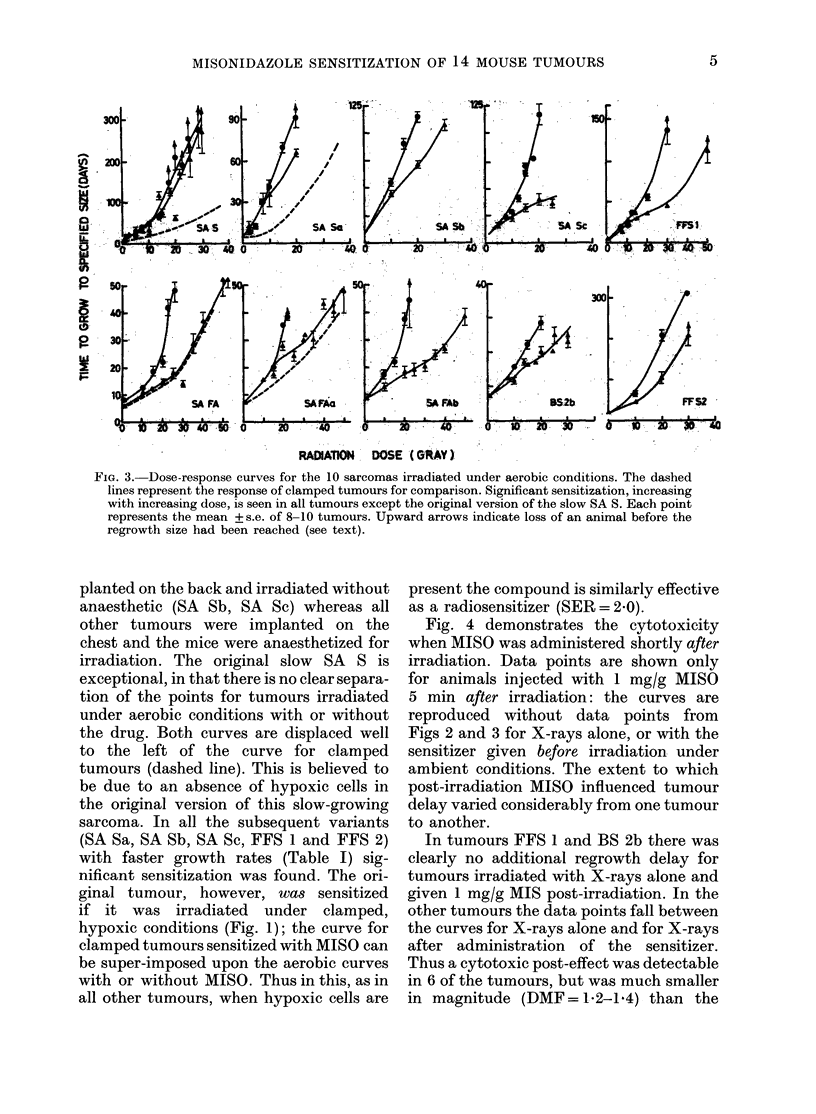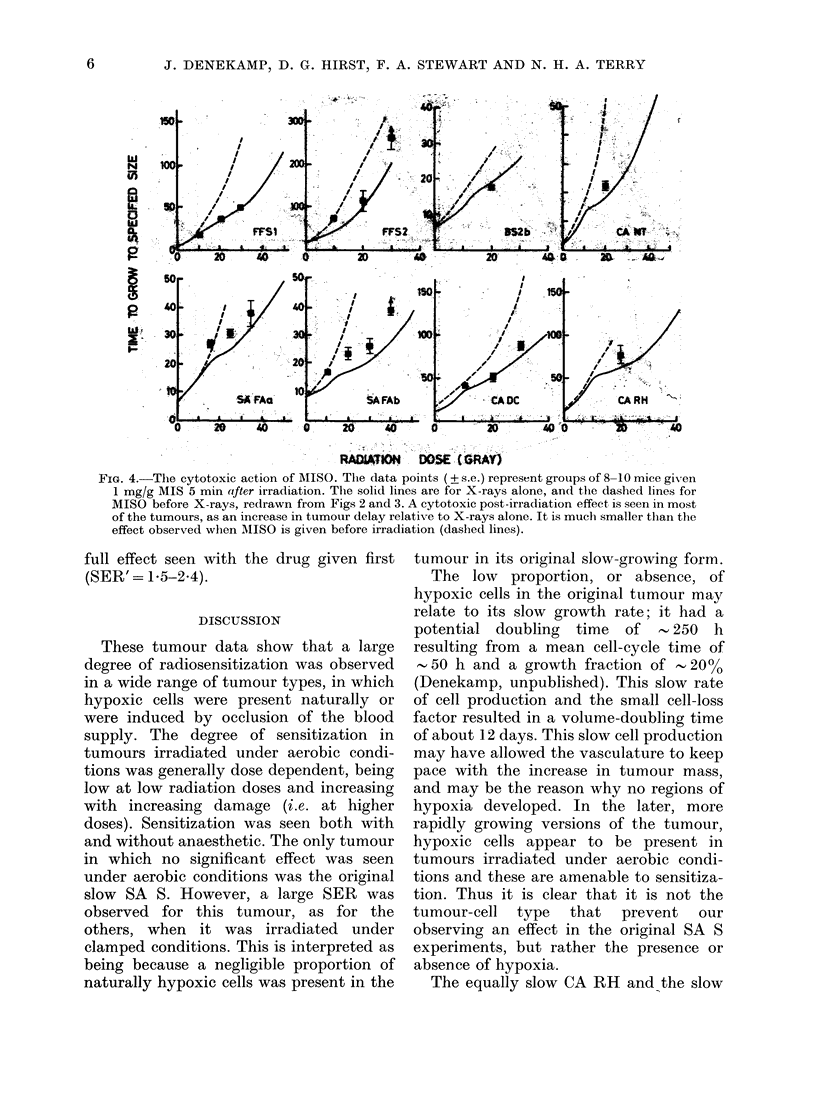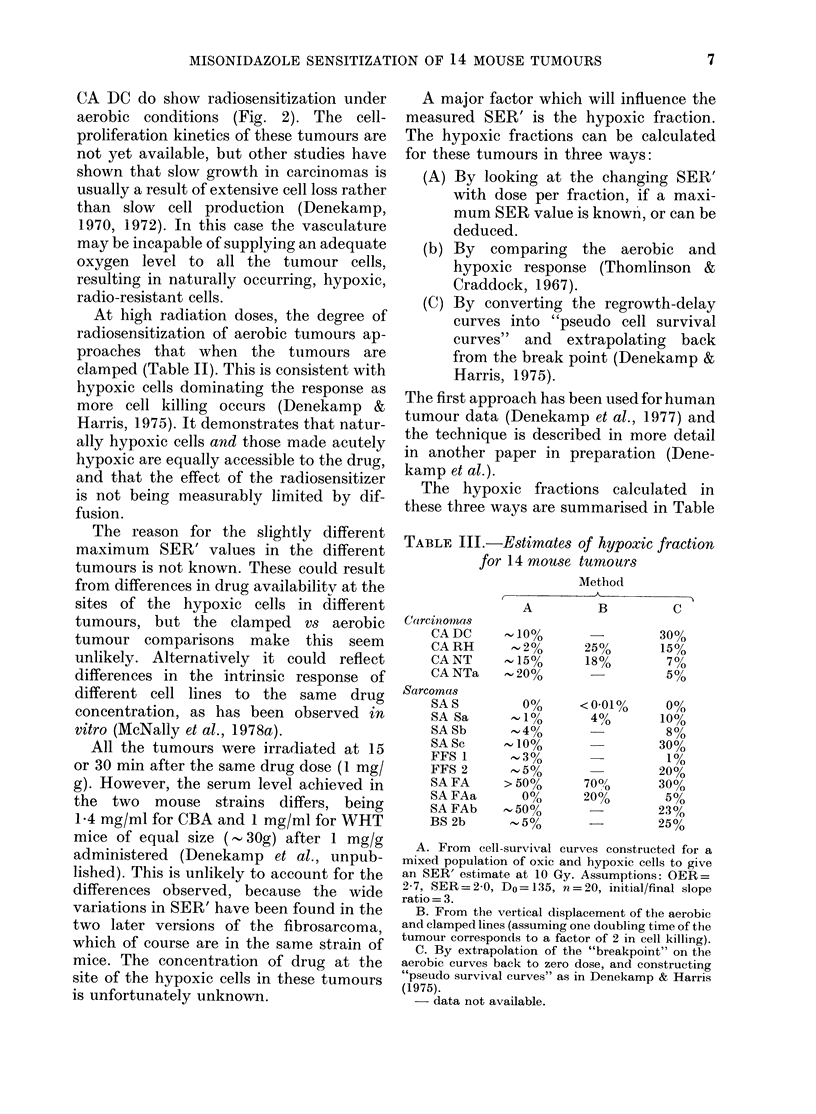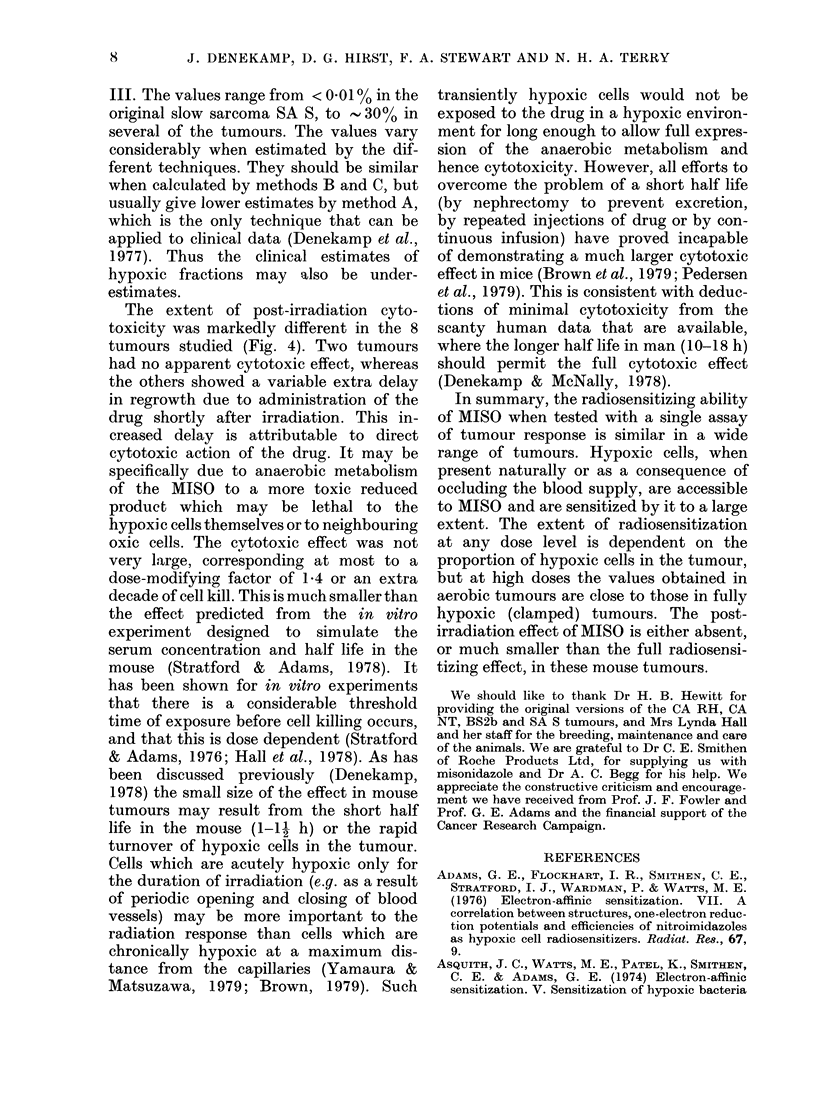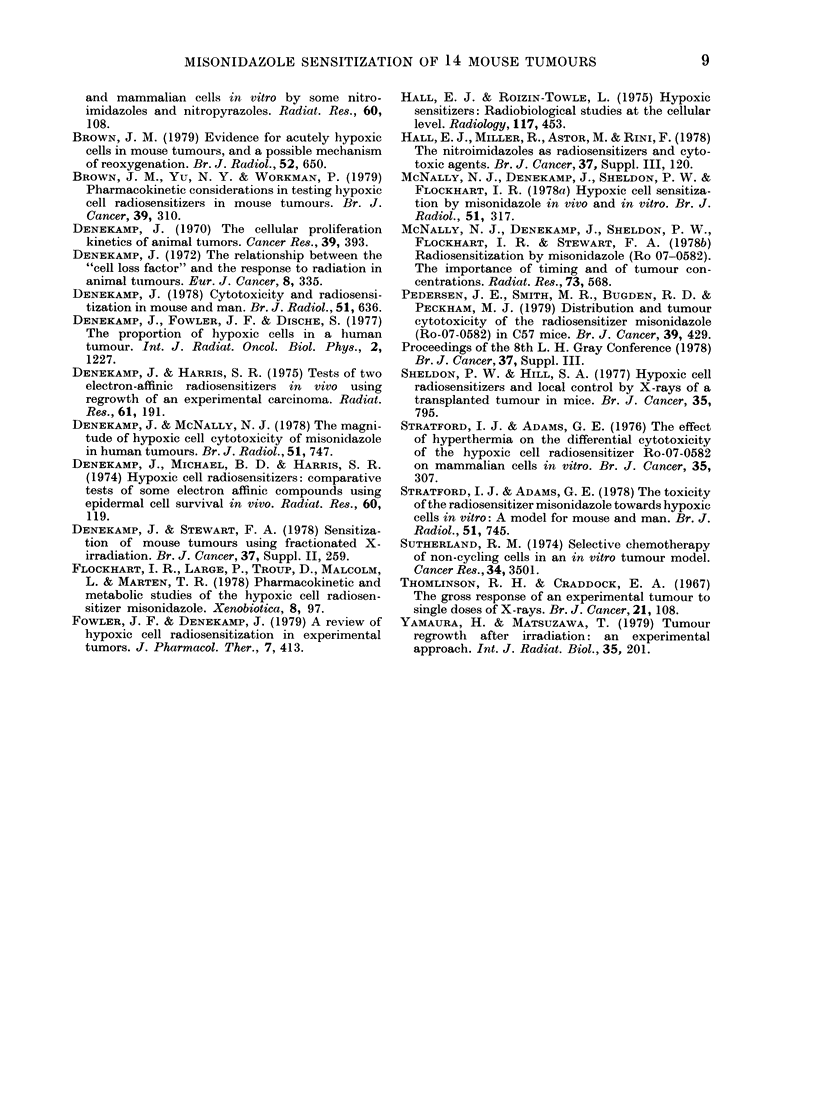# Is tumour radiosensitization by misonidazole a general phenomenon?

**DOI:** 10.1038/bjc.1980.1

**Published:** 1980-01

**Authors:** J. Denekamp, D. G. Hirst, F. A. Stewart, N. H. Terry

## Abstract

The response of 14 mouse tumour sub-lines to the radiosensitizing action of a large single dose of misonidazole (MISO) has been assessed by regrowth delay. In 13 of these, significant enhancement of radiation effect occurred under ambient conditions, indicating sensitization of naturally hypoxic cells. The enhancement observed (SER') varied with the radiation dose, as would be predicted for a mixed oxic/hypoxic cell population. The maximum SER' in these 13 tumours did not depend on histology or regrowth rate. The 14th tumour, a slow-growing sarcoma, was not sensitized under ambient conditions, but showed marked sensitization when clamped to produce acutely hypoxic cells. This is consistent with no hypoxic cells occurring naturally in a sarcoma with a slow rate of growth. Faster-growing variants of this tumour showed radiosensitization under ambient conditions. The slow-growing carcinoma, RH, however, appears to contain hypoxic cells and did show sensitization. The cytotoxic action of MISO was compared with the radiosensitization by administering it after irradiation in 8 of the tumour lines. In 2 tumours no cytotoxicity was observed. In the rest cytotoxicity was significant, but much smaller than the sensitization observed when MISO was administered before irradiation. These regrowth-delay data have been used to calculate hypoxic fractions in 3 ways. Estimates of hypoxic fraction ranged from less than 0.1% in the slow sarcoma to greater than or equal to 30% in several tumours. There is considerable variation in the estimate, according to the technique used.


*Br. J. Cancer* (1980) **41**, 1

### IS TUMOUR RADIOSENSITIZATION BY MISONIDAZOLE A GENERAL PHENOMENON?

J. DENEKAMP, D. G. HIRST, F. A. STEWART AND N. H. A. TERRY

*From the Gray Laboratory of the Cancer Research Campaign, Mount Vernon Hospital, Northwood, Middlesex*

Received 26 July 1979 Accepted 3 September 1979

**Summary.—The response of 14 mouse tumour sub-lines to the radiosensitizing action of a large single dose of misonidazole (MISO) has been assessed by regrowth delay. In 13 of these, significant enhancement of radiation effect occurred under ambient conditions, indicating sensitization of naturally hypoxic cells. The enhancement observed (SER′) varied with the radiation dose, as would be predicted for a mixed oxic/hypoxic cell population. The maximum SER′ in these 13 tumours did not depend on histology or regrowth rate.**

**The 14th tumour, a slow-growing sarcoma, was not sensitized under ambient conditions, but showed marked sensitization when clamped to produce acutely hypoxic cells. This is consistent with no hypoxic cells occurring naturally in a sarcoma with a slow rate of growth. Faster-growing variants of this tumour showed radiosensitization under ambient conditions.**

**The slow-growing carcinoma, RH, however, appears to contain hypoxic cells and did show sensitization.**

**The cytotoxic action of MISO was compared with the radiosensitization by administering it after irradiation in 8 of the tumour lines. In 2 tumours no cytotoxicity was observed. In the rest cytotoxicity was significant, but much smaller than the sensitization observed when MISO was administered before irradiation.**

**These regrowth-delay data have been used to calculate hypoxic fractions in 3 ways. Estimates of hypoxic fraction ranged from <0·1% in the slow sarcoma to ≥30% in several tumours. There is considerable variation in the estimate, according to the technique used.**

THE 2-nitromidazole misonidazole (MISO) has become widely used, both experimentally and in clinical trials, since it was first shown to be an effective radiosensitizer of hypoxic cells *in vitro* and *in vivo* (Asquith *et al.*, 1974; Denekamp *et al.*, 1974). Radiosensitization is a consequence of the compound's electron affinity, and in this way it mimics the effect of oxygen (Asquith *et al.*, 1974; Adams *et al.*, 1976). MISO has been shown to sensitize hypoxic cells *in vitro* but to have no effect on well-oxygenated cells. For this form of radiosensitization the drug must be present at the time of the ionizing event, the degree of radiosensitization being dependent on the local drug concentration (Asquith *et al.*, 1974; McNally *et al.*, 1978*a*).

Studies with experimental tumours, using a variety of assay techniques, have demonstrated that radiosensitization of naturally hypoxic tumour cells can also be achieved *in vitro* (see Proceedings of 8th L. H. Gray Conference, 1978). McNally *et al.* (1978*a*) showed that the same degree of sensitization can be achieved in tumour cells *in vivo*, as can be achieved for Chinese hamster V79 cells *in vitro*, for a range of local drug concentrations. The time between administration of the drug and irradiation may be critical in the mouse, because of competition between delivery of the drug to the tumour cells and the compound's short half life in the mouse ($t_{\frac{1}{2}} = 1–1·5$ h) (Flockhart *et al.*, 1978). The optimum interval varies from 15 to 60 min in different experimental tumours (McNally *et al.*, 1978*b*; Fowler & Denekamp, 1979). The proportion of well-oxygenated to hypoxic cells in the tumour will also influence the observed sensitizer enhancement ratio (SER′) since at low radiation doses the tumour response is dominated by the oxic rather than the hypoxic cells (Denekamp & Harris, 1975; Denekamp *et al.*, 1977).

In addition to the radiosensitizing action of MISO, there is a direct cytotoxic action, which is also specific for hypoxic cells (Sutherland, 1974; Hall & Roizin-Towle, 1975). The magnitude of this effect in human tumours is difficult to predict, but it has been shown to be small compared with the radiosensitization in both mouse and man (Denekamp, 1978; Denekamp & McNally, 1978).

This paper summarizes experimental data on the sensitizing action of MISO on aerobic tumours, assessed using a single assay method (regrowth delay) in 14 different tumour sublines. Several of these tumours have also been studied under conditions of uniform hypoxia, induced by occluding the blood supply with a clamp. In addition, the direct cytotoxic action of the drug has been tested by administering it shortly *after* irradiation in 8 of the tumours. A small proportion of these data has been previously published, but is included here to provide a comprehensive comparison.

### MATERIALS AND METHODS

The regrowth-delay assay and the irradiation procedures which were used in these experiments have been described previously (*e.g.* Denekamp & Harris, 1975; Sheldon & Hill, 1977). The original 6 tumours all arose spontaneously in mice of the CBA/Ht or WHT/Ht strain in our laboratory and have been maintained by s.c. passage in strictly isogeneic mice. The tumour responses are therefore believed to be free of transplantation artefacts. The sarcomas were derived from 3 original spontaneous tumours; differences in their radiation response and/or their growth rates have been observed with successive transplantation (see Table I).

TABLE I.—*Some biological characteristics of the tumours*

| Tumour | Mouse strain | Tumour origin | Volume doubling time (days) | Tumour: Treatment size (mm) | Tumour: Regrowth size (mm) | Year of expts | Treatments used† |
|---|---|---|---|---|---|---|---|
| *Carcinomas* | | | | | | | |
| DA DC | CBA | Spont. 1975 | 10·3 | 8·5 | 12 | 1976–7 | A.B.C |
| CA RH | WHT | Spont. 1966 | 12·0 | 8·5 | 10 | 1976–8 | A,B,C,D,E |
| *CA NT | CBA | Spont. 1968 | 2·8 | 8·5 | 10 | 1973–4 | A,B,C,D,E |
| CA NTa | CBA | From Ca NT 1975 | 2·8 | 8·5 | 10 | 1975–6 | A,B |
| *Sarcomas* | | | | | | | |
| SA S | CBA | Spont. 1962 | 12·4 | 8·5 | 10 | 1975–6 | A,B,D,E |
| SA Sa | CBA | From SAS 1975 | 5·0 | 8·5 | 10 | 1975–6 | A,B,D |
| SA Sb | CBA | From SAS 1977 | 5·8 | 7·5 | 9 | 1977 | A,B |
| SA Sc | CBA | From SAS 1977 | 9·7 | 7·5 | 9 | 1977–8 | A,B |
| FFS 1 | CBA | From SAS 1975 | 3·3 | 8·5 | 12 | 1976 | A,B,C |
| FFS 2 | CBA | From SAS 1975 | 7·8 | 7·5 | 10 | 1976 | A,B,C |
| *SA FA | WHT | Spont. 1973 | 2·7 | 7·5 | 12 | 1974–5 | A,B,D,E |
| *SA FAa | WHT | From SAFA 1976 | 2·8 | 7·5 | 12 | 1976–8 | A,B,C,D |
| ‡SA FAb | WHT | From SAFAa 1978 | 3·8 | 7·5 | 12 | 1978–9 | A,B,C |
| *BS 2b | WHT | Spont. 1967 | 3·5 | 7·5 | 12 | 1974–5 | A,B,C |

* Data previously published in whole or in part (Denekamp & Harris, 1975; McNally *et al.*, 1978b; Denekamp & Stewart, 1978).

† A = X-rays, ambient conditions; B = X-rays after MISO; C = X-rays before MISO; D = X-rays to clamped tumours; E = X-rays to clamped tumours after MISO.

‡ Specific-pathogen-free animals derived by fostering on BSVS mothers, *i.e.* WHTfBv mice.

Tumours were implanted s.c. into batches of 100–250 male mice (aged 2–3 months) and irradiated on a 250 kVp Pantak X-ray set operating at 240 kV, 15mA, with a half-value layer of 1·3 mm Cu and a dose-rate of 2 or 4 gray/min. Unless otherwise stated, the mice were anaesthetized with sodium pentobarbitone, using 60 mg/kg for X-rays alone, or ~40 mg/kg for X-rays plus MISO, because of the combined soporific and toxic effects of the two drugs. Details of the tumour growth rates, the size at irradiation and the size at which regrowth delay was assessed are summarised in Table I. In general sarcomas continued to grow for several days before slowing their growth rate or shrinking (Denekamp, 1972); hence a larger size was often used for the assay of regrowth delay in sarcomas (Table I).

When tumours were clamped for irradiation, to render the cells uniformly hypoxic, this was achieved with a metal D-shaped clamp applied across the base of the tumour at least 10 min before and during irradiation. MISO (kindly provided by Dr C. E. Smithen of Roche Products Ltd, Welwyn Garden City) was always used as a fresh solution, made up in sterile saline at a concentration of 30 mg/ml. One mg/g body weight was administered to each mouse i.p. 15 or 30 min before irradiation or application of the clamp. After treatment the tumours were measured with vernier calipers in 3 dimensions to obtain the geometric mean diameter. The frequency of measurement was decided on the basis of the growth rates, and ranged from 5 times a week for the BS2b tumours to once or twice a week for CA RH and slow SA S.

FIG. 1.—Dose-response curves for tumours clamped to occlude the blood supply and to render all the cells hypoxic. Time to grow from irradiation to the assay size (see Table I for details) is plotted as a function of radiation dose. ▲ = X-rays alone. △ = X-rays 15–30 min after 1 mg/g MISO. All 4 tumours show significant radiosensitization at all dose levels. SER values are indicated against the curves. Each point represents the mean ± s.e.

### RESULTS

The regrowth time from irradiation to a fixed size (see Table I) was measured for each individual tumour. The mean and standard error (s.e.) for each dose group of 8–15 tumours is plotted as a function of radiation dose in Figs 1–4. If an animal was cured or died without the tumour reaching the regrowth size, provided the animal was killed after the mean regrowth time for that dose group, it was included in the analysis, and the error bar has an upward arrow, showing that this is a minimum estimate of regrowth delay.

Fig. 1 shows the response of tumours clamped to make all the cells uniformly hypoxic. Under these conditions a smooth curve was obtained for the tumours treated with X-rays alone, indicating a uniform radioresistance. When 1 mg/g MISO was administered before irradiation a greater radiosensitivity was observed; the curves were displaced to the left. The degree of sensitization can be quantified as an SER (sensitizer enhancement ratio)* by comparing doses to give an equal level of tumour delay without and with the drug. The observed SER′ for aerobic

$*\text{SER} = \frac{\text{dose X-rays without drug}}{\text{dose X-rays with drug}}$ to achieve the same level of radiation effect in fully hypoxic cells.

SER′ = ditto for a mixed population of oxic and hypoxic cells.

TABLE II.—*Sensitization and cytotoxicity of MISO in aerobic and clamped tumours*

| MIS treatment | SER (clamped) 1 mg/g 15 min before clamping | SER′ (aerobic) 1 mg/g 15–30 min before X-rays | DMF (post-effect) 1 mg/g 5 min after irradiation |
|---|---|---|---|
| *Carcinomas* | | | |
| CA DC | — | 1·7 | 1·2 |
| CA RH | 2·0 | 1·8 | 1·4 |
| CA NT | 2·0 | 2·2 | 1·2 |
| CA NTa | — | 2·1 | — |
| *Sarcomas* | | | |
| SA S | 2·0 | <1·1 | — |
| SA Sa | — | 1·3 | — |
| SA Sb | — | 1·6 | — |
| SA Sc | — | 2·0 | — |
| FFS 1 | — | 1·8 | 1·0 |
| FFS 2 | — | 1·5 | ≤1·2 |
| SA FA | 2·2 | 2·0 | — |
| SA FAa | — | 1·8 | 1·2 |
| SA FAb | — | 2·4 | 1·3 |
| BS 2b | — | 1·7 | <1·0 |

The values shown have been derived from the top of each pair of curves in Figs 1–4. — data not available.

FIG. 2.—Dose–response curves for 4 carcinomas irradiated under aerobic conditions. ▲ = X-rays alone. ● = X-rays plus MISO. The dashed lines are for clamped tumours (without drug) from Fig. 1. Each point is the mean ± 1 s.e. of 8–15 tumours. Sensitization is seen in all 4 tumours. The SER′ increases with increasing radiation dose.

tumours is likely to be lower than the maximum SER. Under clamped conditions the SER does not vary much, regardless of dose level, because the cells have all been made hypoxic. Values of SER are indicated in Fig. 1 and summarised in Table II. The SER values are closely similar for the 4 different tumours (2·0–2·2).

Figs 2 and 3 show the results for the 4 carcinomas and the 10 different sarcoma lines irradiated under ambient (unclamped) conditions. In all the tumours except SA S, significant radiosensitization was found when MISO was administered before irradiation. The dashed lines represent the clamped data reproduced without data points.

In Fig. 2 the curves for carcinomas treated with X-rays alone (mice breathing air and with no clamp) are all to some extent biphasic, with "break points" discernible at 10–20 gray. The initial response is believed to be that of the well-oxygenated tumour cells; the response at higher doses becomes progressively dominated by the naturally hypoxic tumour cells, and deviates towards the hypoxic curve, which is reproduced as a dashed line from Fig. 1. The tumours irradiated after MISO administration are more sensitive and give a smooth curve, displaced to the left. SER′ values vary with the level of tumour delay, being greater at higher radiation doses. This is exactly what would be predicted from cell-survival curves for mixed oxic/hypoxic cell populations (*e.g.* Denekamp & Harris, 1975; Denekamp *et al.*, 1977). At the high dose levels the SER′ values range from 1·7 to 2·2 for the 4 carcinomas (Table II).

In Fig. 3 a similar result can be seen for most of the sarcomas. Although the "break points" are not always so prominent, the curves diverge with increasing dose, indicating a larger SER′ at the higher radiation doses. Two of these sets of data were obtained for tumours im-

FIG. 3.—Dose-response curves for the 10 sarcomas irradiated under aerobic conditions. The dashed lines represent the response of clamped tumours for comparison. Significant sensitization, increasing with increasing dose, is seen in all tumours except the original version of the slow SA S. Each point represents the mean ±s.e. of 8–10 tumours. Upward arrows indicate loss of an animal before the regrowth size had been reached (see text).

planted on the back and irradiated without anaesthetic (SA Sb, SA Sc) whereas all other tumours were implanted on the chest and the mice were anaesthetized for irradiation. The original slow SA S is exceptional, in that there is no clear separation of the points for tumours irradiated under aerobic conditions with or without the drug. Both curves are displaced well to the left of the curve for clamped tumours (dashed line). This is believed to be due to an absence of hypoxic cells in the original version of this slow-growing sarcoma. In all the subsequent variants (SA Sa, SA Sb, SA Sc, FFS 1 and FFS 2) with faster growth rates (Table I) significant sensitization was found. The original tumour, however, *was* sensitized if it was irradiated under clamped, hypoxic conditions (Fig. 1); the curve for clamped tumours sensitized with MISO can be super-imposed upon the aerobic curves with or without MISO. Thus in this, as in all other tumours, when hypoxic cells are present the compound is similarly effective as a radiosensitizer (SER = 2·0).

Fig. 4 demonstrates the cytotoxicity when MISO was administered shortly *after* irradiation. Data points are shown only for animals injected with 1 mg/g MISO 5 min *after* irradiation: the curves are reproduced without data points from Figs 2 and 3 for X-rays alone, or with the sensitizer given *before* irradiation under ambient conditions. The extent to which post-irradiation MISO influenced tumour delay varied considerably from one tumour to another.

In tumours FFS 1 and BS 2b there was clearly no additional regrowth delay for tumours irradiated with X-rays alone and given 1 mg/g MIS post-irradiation. In the other tumours the data points fall between the curves for X-rays alone and for X-rays after administration of the sensitizer. Thus a cytotoxic post-effect was detectable in 6 of the tumours, but was much smaller in magnitude (DMF = 1·2–1·4) than the

FIG. 4.—The cytotoxic action of MISO. The data points (±s.e.) represent groups of 8–10 mice given 1 mg/g MIS 5 min *after* irradiation. The solid lines are for X-rays alone, and the dashed lines for MISO before X-rays, redrawn from Figs 2 and 3. A cytotoxic post-irradiation effect is seen in most of the tumours, as an increase in tumour delay relative to X-rays alone. It is much smaller than the effect observed when MISO is given before irradiation (dashed lines).

full effect seen with the drug given first (SER′ = 1·5–2·4).

### DISCUSSION

These tumour data show that a large degree of radiosensitization was observed in a wide range of tumour types, in which hypoxic cells were present naturally or were induced by occlusion of the blood supply. The degree of sensitization in tumours irradiated under aerobic conditions was generally dose dependent, being low at low radiation doses and increasing with increasing damage (*i.e.* at higher doses). Sensitization was seen both with and without anaesthetic. The only tumour in which no significant effect was seen under aerobic conditions was the original slow SA S. However, a large SER was observed for this tumour, as for the others, when it was irradiated under clamped conditions. This is interpreted as being because a negligible proportion of naturally hypoxic cells was present in the tumour in its original slow-growing form.

The low proportion, or absence, of hypoxic cells in the original tumour may relate to its slow growth rate; it had a potential doubling time of ~250 h resulting from a mean cell-cycle time of ~50 h and a growth fraction of ~20% (Denekamp, unpublished). This slow rate of cell production and the small cell-loss factor resulted in a volume-doubling time of about 12 days. This slow cell production may have allowed the vasculature to keep pace with the increase in tumour mass, and may be the reason why no regions of hypoxia developed. In the later, more rapidly growing versions of the tumour, hypoxic cells appear to be present in tumours irradiated under aerobic conditions and these are amenable to sensitization. Thus it is clear that it is not the tumour-cell type that prevent our observing an effect in the original SA S experiments, but rather the presence or absence of hypoxia.

The equally slow CA RH and the slow

CA DC do show radiosensitization under aerobic conditions (Fig. 2). The cell-proliferation kinetics of these tumours are not yet available, but other studies have shown that slow growth in carcinomas is usually a result of extensive cell loss rather than slow cell production (Denekamp, 1970, 1972). In this case the vasculature may be incapable of supplying an adequate oxygen level to all the tumour cells, resulting in naturally occurring, hypoxic, radio-resistant cells.

At high radiation doses, the degree of radiosensitization of aerobic tumours approaches that when the tumours are clamped (Table II). This is consistent with hypoxic cells dominating the response as more cell killing occurs (Denekamp & Harris, 1975). It demonstrates that naturally hypoxic cells *and* those made acutely hypoxic are equally accessible to the drug, and that the effect of the radiosensitizer is not being measurably limited by diffusion.

The reason for the slightly different maximum SER′ values in the different tumours is not known. These could result from differences in drug availability at the sites of the hypoxic cells in different tumours, but the clamped *vs* aerobic tumour comparisons make this seem unlikely. Alternatively it could reflect differences in the intrinsic response of different cell lines to the same drug concentration, as has been observed *in vitro* (McNally *et al.*, 1978*a*).

All the tumours were irradiated at 15 or 30 min after the same drug dose (1 mg/g). However, the serum level achieved in the two mouse strains differs, being 1·4 mg/ml for CBA and 1 mg/ml for WHT mice of equal size (~30g) after 1 mg/g administered (Denekamp *et al.*, unpublished). This is unlikely to account for the differences observed, because the wide variations in SER′ have been found in the two later versions of the fibrosarcoma, which of course are in the same strain of mice. The concentration of drug at the site of the hypoxic cells in these tumours is unfortunately unknown.

A major factor which will influence the measured SER′ is the hypoxic fraction. The hypoxic fractions can be calculated for these tumours in three ways:

(A) By looking at the changing SER′ with dose per fraction, if a maximum SER value is known, or can be deduced.

(b) By comparing the aerobic and hypoxic response (Thomlinson & Craddock, 1967).

(C) By converting the regrowth-delay curves into "pseudo cell survival curves" and extrapolating back from the break point (Denekamp & Harris, 1975).

The first approach has been used for human tumour data (Denekamp *et al.*, 1977) and the technique is described in more detail in another paper in preparation (Denekamp *et al.*).

The hypoxic fractions calculated in these three ways are summarised in Table

TABLE III.—*Estimates of hypoxic fraction for* 14 *mouse tumours*

| | Method | | |
|---|---|---|---|
| | A | B | C |
| *Carcinomas* | | | |
| CA DC | ~10% | — | 30% |
| CA RH | ~2% | 25% | 15% |
| CA NT | ~15% | 18% | 7% |
| CA NTa | ~20% | — | 5% |
| *Sarcomas* | | | |
| SA S | 0% | <0·01% | 0% |
| SA Sa | ~1% | 4% | 10% |
| SA Sb | ~4% | — | 8% |
| SA Sc | ~10% | — | 30% |
| FFS 1 | ~3% | — | 1% |
| FFS 2 | ~5% | — | 20% |
| SA FA | >50% | 70% | 30% |
| SA FAa | 0% | 20% | 5% |
| SA FAb | ~50% | — | 23% |
| BS 2b | ~5% | — | 25% |

A. From cell-survival curves constructed for a mixed population of oxic and hypoxic cells to give an SER′ estimate at 10 Gy. Assumptions: OER = 2·7, SER = 2·0, $D_0$ = 135, $n$ = 20, initial/final slope ratio = 3.

B. From the vertical displacement of the aerobic and clamped lines (assuming one doubling time of the tumour corresponds to a factor of 2 in cell killing).

C. By extrapolation of the "breakpoint" on the aerobic curves back to zero dose, and constructing "pseudo survival curves" as in Denekamp & Harris (1975).

— data not available.

III. The values range from $< 0.01\%$ in the original slow sarcoma SA S, to $\sim 30\%$ in several of the tumours. The values vary considerably when estimated by the different techniques. They should be similar when calculated by methods B and C, but usually give lower estimates by method A, which is the only technique that can be applied to clinical data (Denekamp *et al.*, 1977). Thus the clinical estimates of hypoxic fractions may also be underestimates.

The extent of post-irradiation cytotoxicity was markedly different in the 8 tumours studied (Fig. 4). Two tumours had no apparent cytotoxic effect, whereas the others showed a variable extra delay in regrowth due to administration of the drug shortly after irradiation. This increased delay is attributable to direct cytotoxic action of the drug. It may be specifically due to anaerobic metabolism of the MISO to a more toxic reduced product which may be lethal to the hypoxic cells themselves or to neighbouring oxic cells. The cytotoxic effect was not very large, corresponding at most to a dose-modifying factor of 1·4 or an extra decade of cell kill. This is much smaller than the effect predicted from the *in vitro* experiment designed to simulate the serum concentration and half life in the mouse (Stratford & Adams, 1978). It has been shown for *in vitro* experiments that there is a considerable threshold time of exposure before cell killing occurs, and that this is dose dependent (Stratford & Adams, 1976; Hall *et al.*, 1978). As has been discussed previously (Denekamp, 1978) the small size of the effect in mouse tumours may result from the short half life in the mouse (1–1½ h) or the rapid turnover of hypoxic cells in the tumour. Cells which are acutely hypoxic only for the duration of irradiation (*e.g.* as a result of periodic opening and closing of blood vessels) may be more important to the radiation response than cells which are chronically hypoxic at a maximum distance from the capillaries (Yamaura & Matsuzawa, 1979; Brown, 1979). Such transiently hypoxic cells would not be exposed to the drug in a hypoxic environment for long enough to allow full expression of the anaerobic metabolism and hence cytotoxicity. However, all efforts to overcome the problem of a short half life (by nephrectomy to prevent excretion, by repeated injections of drug or by continuous infusion) have proved incapable of demonstrating a much larger cytotoxic effect in mice (Brown *et al.*, 1979; Pedersen *et al.*, 1979). This is consistent with deductions of minimal cytotoxicity from the scanty human data that are available, where the longer half life in man (10–18 h) should permit the full cytotoxic effect (Denekamp & McNally, 1978).

In summary, the radiosensitizing ability of MISO when tested with a single assay of tumour response is similar in a wide range of tumours. Hypoxic cells, when present naturally or as a consequence of occluding the blood supply, are accessible to MISO and are sensitized by it to a large extent. The extent of radiosensitization at any dose level is dependent on the proportion of hypoxic cells in the tumour, but at high doses the values obtained in aerobic tumours are close to those in fully hypoxic (clamped) tumours. The post-irradiation effect of MISO is either absent, or much smaller than the full radiosensitizing effect, in these mouse tumours.

We should like to thank Dr H. B. Hewitt for providing the original versions of the CA RH, CA NT, BS2b and SA S tumours, and Mrs Lynda Hall and her staff for the breeding, maintenance and care of the animals. We are grateful to Dr C. E. Smithen of Roche Products Ltd, for supplying us with misonidazole and Dr A. C. Begg for his help. We appreciate the constructive criticism and encouragement we have received from Prof. J. F. Fowler and Prof. G. E. Adams and the financial support of the Cancer Research Campaign.

### REFERENCES

Adams, G. E., Flockhart, I. R., Smithen, C. E., Stratford, I. J., Wardman, P. & Watts, M. E. (1976) Electron-affinic sensitization. VII. A correlation between structures, one-electron reduction potentials and efficiencies of nitroimidazoles as hypoxic cell radiosensitizers. *Radiat. Res.*, **67**, 9.

Asquith, J. C., Watts, M. E., Patel, K., Smithen, C. E. & Adams, G. E. (1974) Electron-affinic sensitization. V. Sensitization of hypoxic bacteria and mammalian cells *in vitro* by some nitroimidazoles and nitropyrazoles. *Radiat. Res.*, **60**, 108.

BROWN, J. M. (1979) Evidence for acutely hypoxic cells in mouse tumours, and a possible mechanism of reoxygenation. *Br. J. Radiol.*, **52**, 650.

BROWN, J. M., YU, N. Y. & WORKMAN, P. (1979) Pharmacokinetic considerations in testing hypoxic cell radiosensitizers in mouse tumours. *Br. J. Cancer*, **39**, 310.

DENEKAMP, J. (1970) The cellular proliferation kinetics of animal tumors. *Cancer Res.*, **39**, 393.

DENEKAMP, J. (1972) The relationship between the "cell loss factor" and the response to radiation in animal tumours. *Eur. J. Cancer*, **8**, 335.

DENEKAMP, J. (1978) Cytotoxicity and radiosensitization in mouse and man. *Br. J. Radiol.*, **51**, 636.

DENEKAMP, J., FOWLER, J. F. & DISCHE, S. (1977) The proportion of hypoxic cells in a human tumour. *Int. J. Radiat. Oncol. Biol. Phys.*, **2**, 1227.

DENEKAMP, J. & HARRIS, S. R. (1975) Tests of two electron-affinic radiosensitizers *in vivo* using regrowth of an experimental carcinoma. *Radiat. Res.*, **61**, 191.

DENEKAMP, J. & MCNALLY, N. J. (1978) The magnitude of hypoxic cell cytotoxicity of misonidazole in human tumours. *Br. J. Radiol.*, **51**, 747.

DENEKAMP, J., MICHAEL, B. D. & HARRIS, S. R. (1974) Hypoxic cell radiosensitizers: comparative tests of some electron affinic compounds using epidermal cell survival *in vivo*. *Radiat. Res.*, **60**, 119.

DENEKAMP, J. & STEWART, F. A. (1978) Sensitization of mouse tumours using fractionated X-irradiation. *Br. J. Cancer*, **37**, Suppl. II, 259.

FLOCKHART, I. R., LARGE, P., TROUP, D., MALCOLM, L. & MARTEN, T. R. (1978) Pharmacokinetic and metabolic studies of the hypoxic cell radiosensitizer misonidazole. *Xenobiotica*, **8**, 97.

FOWLER, J. F. & DENEKAMP, J. (1979) A review of hypoxic cell radiosensitization in experimental tumors. *J. Pharmacol. Ther.*, **7**, 413.

HALL, E. J. & ROIZIN-TOWLE, L. (1975) Hypoxic sensitizers: Radiobiological studies at the cellular level. *Radiology*, **117**, 453.

HALL, E. J., MILLER, R., ASTOR, M. & RINI, F. (1978) The nitroimidazoles as radiosensitizers and cytotoxic agents. *Br. J. Cancer*, **37**, Suppl. III, 120.

MCNALLY, N. J., DENEKAMP, J., SHELDON, P. W. & FLOCKHART, I. R. (1978*a*) Hypoxic cell sensitization by misonidazole *in vivo* and *in vitro*. *Br. J. Radiol.*, **51**, 317.

MCNALLY, N. J., DENEKAMP, J., SHELDON, P. W., FLOCKHART, I. R. & STEWART, F. A. (1978*b*) Radiosensitization by misonidazole (Ro 07–0582). The importance of timing and of tumour concentrations. *Radiat. Res.*, **73**, 568.

PEDERSEN, J. E., SMITH, M. R., BUGDEN, R. D. & PECKHAM, M. J. (1979) Distribution and tumour cytotoxicity of the radiosensitizer misonidazole (Ro-07-0582) in C57 mice. *Br. J. Cancer*, **39**, 429.

Proceedings of the 8th L. H. Gray Conference (1978) *Br. J. Cancer*, **37**, Suppl. III.

SHELDON, P. W. & HILL, S. A. (1977) Hypoxic cell radiosensitizers and local control by X-rays of a transplanted tumour in mice. *Br. J. Cancer*, **35**, 795.

STRATFORD, I. J. & ADAMS, G. E. (1976) The effect of hyperthermia on the differential cytotoxicity of the hypoxic cell radiosensitizer Ro-07-0582 on mammalian cells *in vitro*. *Br. J. Cancer*, **35**, 307.

STRATFORD, I. J. & ADAMS, G. E. (1978) The toxicity of the radiosensitizer misonidazole towards hypoxic cells *in vitro*: A model for mouse and man. *Br. J. Radiol.*, **51**, 745.

SUTHERLAND, R. M. (1974) Selective chemotherapy of non-cycling cells in an *in vitro* tumour model. *Cancer Res.*, **34**, 3501.

THOMLINSON, R. H. & CRADDOCK, E. A. (1967) The gross response of an experimental tumour to single doses of X-rays. *Br. J. Cancer*, **21**, 108.

YAMAURA, H. & MATSUZAWA, T. (1979) Tumour regrowth after irradiation: an experimental approach. *Int. J. Radiat. Biol.*, **35**, 201.